# Identification of PLCγ2 activators for the treatment of Alzheimer’s disease

**DOI:** 10.1002/alz70859_103518

**Published:** 2025-12-25

**Authors:** Brent Clayton, Steven M Massey, Shaoyou Chu, Emily R Mason, Stephanie J Bissel, Logan M Bedford, Stacey J Sukoff Rizzo, Andrew D. Mesecar, Bridget L Kaiser, Emma K Lendy, Bruce T. Lamb, Alan D. Palkowitz, Timothy I. Richardson

**Affiliations:** ^1^ Indiana University School of Medicine, Indianapolis, IN USA; ^2^ Indiana University, Indianapolis, IN USA; ^3^ University of Pittsburgh School of Medicine, Pittsburgh, PA USA; ^4^ Purdue University, West Lafayette, IN USA; ^5^ Purdue, West Lafayette, IN USA; ^6^ Indiana Biosciences Research Institute, Indianapolis, IN USA

## Abstract

**Background:**

The role of microglia in neuroinflammation is widely recognized as a key contributor to the pathogenesis of Alzheimer’s disease (AD). Genome‐wide association studies have identified PLCγ2 as a key contributor, with specific variants conferring either risk or protection. Notably, the protective PLCγ2•P522R variant is associated with increased mRNA expression, protein levels, and innate activity, whereas the risk variant PLCγ2•M28L shows the opposite trend. Based on these findings, we hypothesize that small molecules capable of enhancing PLCγ2 expression or directly activating the protein could mimic the protective effects of the P522R variant. Such an approach may represent a promising therapeutic strategy to mitigate disease progression and cognitive decline in AD patients.

**Method:**

We performed high‐throughput screening including DNA Encoded Library (DEL) and Affinity Selection Mass Spectrometry (ASMS) using full‐length protein to identify novel small molecules which bind to PLCγ2. Target engagement was confirmed using Differential Scanning Fluorimetry (DSF) and Cellular Thermal Shift Assay (CETSA). Structure activity relationship (SAR) studies were performed to synthesize analogs and optimize for binding and cellular pharmacology in IP‐One and phagocytosis assays. Top compounds have been studied in vivo to assess pharmacokinetic properties as well as impact on neuroinflammation.

**Result:**

Novel PLCγ2 activators have been discovered and preliminary optimization has been completed. These compounds have shown positive results for target engagement, biochemical activity, and cellular pharmacology. In silico predictions indicated the molecule structures are suitable CNS drug discovery program starting points.

**Conclusion:**

Activation of PLCγ2 is a novel therapeutic strategy for treatment of AD. We identified structurally distinct molecular scaffolds capable of enzyme activation and cellular activity. Recommendations for use of probe molecules in target validation studies and the development of lead‐like molecules for clinical studies will be made.

